# Queen dominance and worker policing control reproduction in a threatened ant

**DOI:** 10.1186/1472-6785-11-21

**Published:** 2011-09-30

**Authors:** Jürgen Trettin, Monika Haubner, Alfred Buschinger, Jürgen Heinze

**Affiliations:** 1 Biologie I, Universität Regensburg, Universitätsstr. 31, Regensburg, 93053, Germany; 2 Rossbergring 18, Reinheim, 64354, Germany

## Abstract

**Background:**

Efficient division of reproductive labor is a crucial characteristic of social insects and underlies their ecological and evolutionary success. Despite of the harmonious appearance of insect societies, nestmates may have different interests concerning the partitioning of reproduction among group members. This may lead to conflict about reproductive rights. As yet, few studies have investigated the allocation of reproduction among queens in multi - queen societies ("reproductive skew"). In the ant *Leptothorax acervorum*, reproductive skew varies considerably among populations. While reproduction is quite equally shared among nestmate queens in most populations from boreal Eurasia (low skew), colonies from populations at the edge of the species' range are characterized by "functional monogyny," i.e., high skew. The proximate mechanisms underlying high skew, in particular how workers influence which queen lays eggs, are not well understood. We investigated the behavior of queens and workers in functionally monogynous colonies of *L. acervorum *from two mountain ranges in central Spain.

**Results:**

We provide evidence for both queen and worker influence on the outcome of conflict over reproduction in colonies of *L. acervorum *from Spain. The patterns of queen - queen aggression and worker - queen grooming and feeding after hibernation allowed predicting, which queen later began to lay eggs. In contrast, worker aggression towards queens was not clearly associated with a queen's future reproductive success. Queen - queen and worker - queen aggression differed in quality: queens typically engaged in ritualized dominance behavior, such as antennal boxing, while workers also attacked queens by biting and prolonged pulling on their legs and antennae. In several cases, overt worker aggression led to the expulsion of queens from the nest or their death.

**Conclusion:**

We conclude that queens of *L. acervorum *from Spain establish rank orders by ritualized dominance interactions, such as antennal boxing. Workers may reinforce these hierarchies by preferentially feeding and grooming high ranking queens and attacking lower ranking queens. Aggressive worker policing may thus stabilize functional monogyny. Optimal skew models predict that high skew in ants is associated with high dispersal costs. In central Spain, *L. acervorum *is restricted to small patches at higher elevations, which presumably makes dispersal and colony founding difficult. Because of the ecological requirements of *L. acervorum *and the predicted large impact of global change on central Spain, the functionally monogynous populations of this ant must be considered as threatened.

## Background

Efficient division of reproductive labor is one of the key characteristics of social insects (e.g., honeybees, ants, and wasps) and underlies their enormous evolutionary success. Despite of the harmonious appearance of their societies, who reproduces and who does not is often controversial, and how conflict about reproductive rights is resolved has become focus of both theoretical and empirical research [1-4].

Punishment, policing, and dominance regulate egg laying in societies in which all females are morphologically identical and potentially capable of reproducing [4,5]. In species with a clear queen-worker diphenism, workers normally refrain from laying eggs in response to the odor of a fertile queen [6,7], presumably because they otherwise risk to be attacked by their nestmates. Worker altruism in insect societies therefore can be considered to be "enforced" at least in part [8].

Comparatively little is known about another type of conflict, the one about the partitioning of reproduction when colonies contain multiple queens. In facultatively polygynous species, egg laying rates and genetic maternity assignments usually suggest a more or less equal contribution of queens to the egg pile and queens do not interact aggressively ("low reproductive skew"; [9-13]). Brood from individual queens may differ in its propensity to develop into sexuals, but this is not due to social interactions among queens [14].

High reproductive skew, i.e., a highly unequal partitioning of reproduction, has as yet been described for only a handful of species ("functional monogyny"; [9]). For example, in *Leptothorax gredleri *and related species, nestmate queens violently antennate and bite one another and form social hierarchies, in which only the top - ranking queen begins to reproduce [15-18]. At a later stage, subordinate queens may be attacked and expelled by workers [15-18]. This resembles the elimination of surplus queens in founding associations [19,20] and polygynous species (e.g., [17]). In accordance with models of optimal skew [21], functional monogyny is associated with patchy habitat in which solitary nest founding is costly [22].

In the Holarctic ant *Leptothorax acervorum*, reproductive skew appears to vary with habitat characteristics. *L. acervorum *is widely distributed over large parts of the northern hemisphere [23,24]. Colonies are facultatively polygynous in the extended coniferous forests of Central Europe and Siberia [9-13], but functionally monogynous where they are only patchily distributed, i.e., on sun-exposed slopes in Alaska, in light clearings in Hokkaido, and at the southern limit of its range in mountainous areas in central Spain [18,25-28].

Functional monogyny appears to be based on queen dominance interactions and fighting in colonies from Alaska [25] and Hokkaido [18]. However, a recent study suggested that queen-queen interactions are rare in the population from central Spain and that instead worker aggression regulates which queen may lay eggs [29]. Here, we document that both queen dominance and worker policing contribute to the regulation of reproduction in *L. acervorum *from central Spain.

## Results

Details on the origin and composition of colonies used in this study are given in Table 1. All queens (n = 35) used in the analysis had sperm in their spermathecae. As expected from previous dissections, in each colony only a single queen had fully active ovaries with elongated ovarioles and corpora lutea. Several other queens had partly elongated ovarioles showing traces of previous, temporary egg production. However, their ovaries appeared to have reverted to an inactive state (Table 2). The colonies SA 03 and SA 88 were observed (Table 1) but their queens could not be dissected. Consequently, both colonies were removed from statistical analysis.

**Table 1:** Location, composition and observation time for each colony.

Site	Colony	Location	Altitude	**No**.	**No**.	Observation
			(in m)	Queens	Workers	time (in hr)
S^ra ^de	SA 03	40.52506°, -1.64692°	1718	5	30 ± 10	8.4
Albarracin	SA 20	40.49877°, -1.59101°	1667	4	25	9.7
	SA 51	40.49877°, -1.59101°	1667	4	30	7.0
	SA 68	40.59878°, -1.71198°	1683	6	40	8.8
	SA 88	40.59878°, -1.71198°	1683	5	50 ± 10	7.5
	SA 102	40.49877°, -1.59101°	1667	6	15	15.9
	SA 109	40.49877°, -1.59101°	1667	7	20	26.8
	SA 125	40.59878°, -1.71198°	1683	4	25	10.0
	SA 151	40.52447°, -1.64120°	1657	6	20	27.9

S^ra ^de	SG 04	40.37121°, -0.62730°	1959	5	40	15.4
Gúdar	SG 32	40.39070°, -0.66517°	2014	4	15	24.8
	SG 40	40.38626°, -0.64362°	1958	5	30	20.4

**Table 2:** Mating and reproductive status of queens for all colonies used in the analysis

	No. Queens			No. Queens	
Colony	Mated	Unmated	UD	Reproductive	Non-reproductive
SA 20	4	0	0	**2**	2
SA 51	2	0	2	1	3
SA 68	6	0	0	1	5
SA 102	2	3	1	1	5
SA 109	5	1	1	1	6
SA 125	3	1	0	1	3
SA 151	4	2	0	1	5
SG 04	3	1	1	1	4
SG 32	3	1	0	1	3
SG 40	4	1	0	1	4

Data indicated as the number of queens per category and colony. With the exception of colony SA 20, all colonies had one reproductive queen. As colony SA 20 was observed only for a rather short period (9.7 h over 10 days), the reproductive hierarchy, and hence functional monogyny, could not be fully established. The mating status of several queens that were killed before dissection could not be determined (UD).

**Figure 1:**
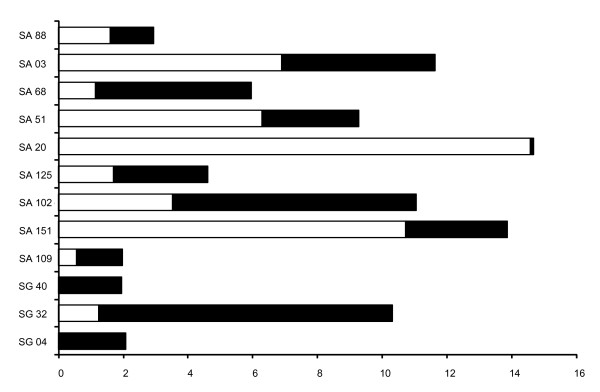
**Aggression among queens (white bars) and between workers and queens (black bars) in colonies of the ant *Leptothorax acervorum *from central Spain**. Aggression is shown in attacks h^-1^.

**Figure 2:**
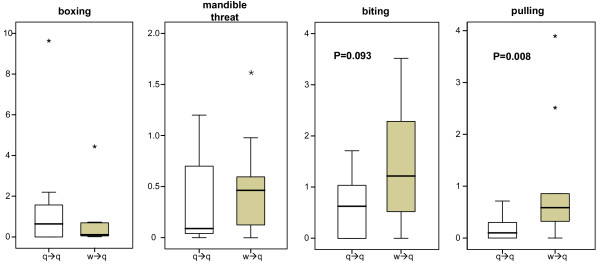
**Differences in the type of aggressive behavior between workers and queens**. Queens show significantly less pulling behavior than workers (N = 10 colonies). Worker - queen aggression (w→q) is shown as grey boxes and queen - queen aggression (q→q) as white boxes. Outliers are indicated as *.

Casual observations had already indicated queen - queen aggression directly after collecting in fall. More detailed studies after artificial hibernation corroborated this result: aggressive interactions among queens occurred in 11 out of 12 studied colonies from S^ra ^de Albarracin and S^ra ^de Gúdar (Figure 1 and Additional File 1). In all colonies, we in addition observed worker aggression towards queens. In total, queens received 53% of attacks from other queens (median and quartiles per queen 0.12, 0.0, 0.9 attacks per hour). Workers were responsible for 47% of the attacks toward queens (median and quartiles per queen: 0.36, 0.12, 1.16 attacks per hour). In addition to the antagonistic behavior, we also observed sociopositive interactions (grooming and feeding). The observed level of grooming and trophallaxis received by queens ranged from zero to 2.75 events h^-1 ^(median, quartiles per queen: 0.7, 0.44, 1.33 acts per hour). The quality of queen - queen and worker - queen antagonism differed considerably. Queens were significantly more often pulled by workers than by other queens (Figure 2, Mann - Whitney U test: *U *= 16, *N*_*1 *_= *N*_*2 *_= 10, *P *= 0.008). We found a similar trend for differences in biting (Figure 2, *U *= 27.5, *P *= 0.093). In contrast, there was no significant difference between queens and workers in the frequency of antennal boxing (Figure 2, *U *= 43, *P *= 0.61) and mandible threats (Figure 2, *U *= 43, *P *= 0.63). In numerous instances we observed several workers simultaneously pulling on the antennae or legs of a queen. This severe pulling occasionally led to expulsion of queens and to the death of three queens from three colonies.

Statistical analysis showed that, over all colonies, future reproductive status of queens was not reliably predicted by worker - queen aggression (logistic regression: likelihood - ratio - Chi^2 ^= 0.05, d. f. = 1, P = 0.83, 31 queens), but instead by the frequency of sociopositive acts from workers to queens (logistic regression: likelihood - ratio - Chi^2 ^= 11.42, d. f. = 1, P < 0.001, 35 queens). Furthermore, the individual average dominance index (ADI, [30]) of each queen calculated from queen - queen aggression predicted the future reproductive status of a queen (likelihood - ratio - Chi^2 ^= 4.50, d. f. = 1, P = 0.034, 29 queens). These results are corroborated by Scheirer - Ray - Hare tests: worker - queen aggression was not associated with the queens' future reproductive status (H = 0.06, d. f. = 1, P = 0.81) in contrast to worker - queen grooming and trophallaxis (H = 5.61, d. f. = 1, P = 0.018) and, although at only marginal significance, ADI (H = 3.03, d. f. = 1, P = 0.08). Both queen aggression (8 out of 560 attacks) and worker aggression (16 out of 572 attacks) led to the expulsion of subordinate queens.

Across all five microsatellite loci, average relatedness of nestmate workers from 10 colonies from S^ra ^de Albarracin was 0.67 ± SE 0.06. A linkage analysis revealed significant linkage disequilibrium between the loci LXAGA1 and Myrt3 (P = 0.005). Therefore, we repeated the relatedness analysis without Myrt3 and obtained a relatedness coefficient of 0.66 ± 0.05. Both values are not significantly less than the value expected for full sisters (0.75; t = -1.489, P = 0.17 vs. t = -1.592, P = 0.15). The inbreeding coefficient (F = 0.10 ± 0.08) was not significantly different from zero (t = 1.25, P > 0.2).

## Discussion

Our study provides evidence for the regular occurrence of aggressive interactions among queens in functionally monogynous colonies of the ant *L. acervorum *from central Spain. Queen - queen aggression leads to the establishment of rank orders, in which the top - ranking queen becomes fertile. Queen dominance behavior, together with additional, unevenly distributed aggressive or sociopositive actions of workers, proximately underlie high reproductive skew and lead to functional monogyny.

Both, the frequency of queen - queen aggression and of grooming and feeding behavior of queens by workers predicted which queen later started to lay eggs. Future reproductive queens typically showed a high level of aggression towards other queens and were frequently groomed and fed by workers. In contrast to queen antagonism, worker aggression towards queens was not associated with future reproductive status. Worker aggression was considerably more overtly aggressive than queen aggression.

Concerning the mechanisms underlying functional monogyny, *L. acervorum *from central Spain thus is similar to other functionally monogynous ants. Similar aggressive interactions among mated queens were observed previously in *L. acervorum *from Japan [18] and Alaska [26], and functionally monogynous *Leptothorax *sp. A, *L. gredleri*, and *Formicoxenus provancheri *[15,16,31]. In all this cases, antagonistic behavior among queens after hibernation contributes to the establishment of dominance hierarchies in which only the highest - ranking individual is reproducing. Queen - queen aggression is commonly complemented by discriminatory treatment of queens by workers. Worker behavior appears to accentuate rank differences, in that workers feed and groom dominant queens more frequently and attack, expel or even kill subordinate queens. For example, subordinate queens of *Leptothorax *sp. A and *L. gredleri *are attacked by workers when reentering the nest after having been expelled by dominant queens [15,16]. Similarly, both queen - queen and

worker - queen aggression have been observed in functionally monogynous colonies of *L. acervorum *from Japan and Alaska [18,26]. It is therefore not surprising that worker aggression occurs in *L. acervorum *from Spain. It serves to prevent surplus queens from becoming reproductive [17] and can thus be considered as policing (e.g., [29,32]).

Workers therefore play an important role in the regulation of reproduction, but presumably only after the queens themselves have established rank orders. As suggested by Gill and Hammond [29], workers may act as "agents" of the dominant queen, in that they eliminate rival queens once the hierarchy has formed. Queens of functionally monogynous *L. gredleri *besmear their opponents with Dufour gland secretions, which elicit aggression from workers [16,33]. Similar "punishment" is known from the queenless ant *Dinoponera quadriceps *[34] and the fighting males of *Cardiocondyla *[35]. Queen besmearing has not been observed in *L. acervorum *from Spain, but workers might use more subtle signals or chemical differences among queens to discriminate dominant and subordinate queens. Genetic data suggest that at least in *L. gredleri*, queens may seek adoption into an alien colony and usurp the top-position in the hierarchy [J.H., unpublished]. Workers appear to support the queen that first becomes reproductive and not necessarily their own mother, similar to the situation in founding associations [19].

Colonies of *L. acervorum *from Central, Western, and Northern Europe are facultatively polygynous (e.g. [9-13]), i.e., the species is functionally monogynous in the periphery of its geographic range. This matches predictions from skew models: marginal areas are suboptimal, and suitable sites for colony founding and nesting are limited. Therefore, such marginal or patchy habitats should favor the development of multiple - queening with less dispersal, higher queen relatedness, higher aggression and higher skew in ant colonies. [21,22]. In the mountains of central Spain, *L. acervorum *are restricted to humid areas in pine dominated forests at elevations above 1500 m [27]. Though we did not map the location of colonies in detail, colonies appeared to be restricted to relatively small patches surrounded by large, unpopulated areas. Given that the Sierras of central Spain are not much higher than 2000 m and expected to be extremely affected by global change [36], it is likely that such patches will become less and less common. Even though functionally monogynous colonies have been found in more northern mountain ranges (JT unpublished), the Spanish high-skew populations of *L. acervorum *are probably highly threatened, in contrast to the wide - ranging low - skew populations of boreal Eurasia.

The results of our behavioral study stand in marked contrast to previous observations that worker - queen aggression rather than queen - queen antagonism underlies high reproductive skew [29]. At present we can only speculate about the cause for this discrepancy. First, our study revealed considerable variation in the occurrence of queen - queen aggression among colonies. There may be subtle differences in ecological, behavioral, or genetic properties between the colonies studied by Gill and Hammond [29] and those in our study. Second, and more importantly, the two studies differ in several critical aspects. The behavioral analysis of Gill and Hammond [29] apparently did not include antennal boxing, but focused on more violent aggression, which, as we show above, is more commonly exhibited by workers. Furthermore, they subjected the ants to only six weeks of artificial hibernation. This is much shorter than natural winter and also shorter than the standard hibernation time established for laboratory cultures of *Leptothorax *ants by Buschinger [37]. Conditions in S^ra ^de Albarracin and S^ra ^de Gúdar are harsh and temperatures can fall below 0°C during seven months or longer [27]. *Leptothorax *are active at nest temperatures of 5°C for several weeks after the onset of hibernation [38]. Young queens begin to establish hierarchies after mating in late summer and fall [39, see also our study] and a period of six weeks is probably too short to obscure rank differences among queens.

## Conclusion

Our study documents that aggression among nestmate queens occurs in colonies of two functionally monogynous populations of *L. acervorum *from central Spain. Queen - queen aggression contributes significantly to the formation of reproductive hierarchies among queens and ultimately to the high reproductive skew in these populations.

In addition, we could show that workers influence skew, especially by differentially feeding and grooming queens. In contrast, the frequency of worker - queen aggression was not associated with a queen's future reproductive success, though worker aggression is certainly involved in later eliminating subordinate queens or driving them out of the nest. The intraspecific variation in reproductive skew makes *L. acervorum *a valuable model system to investigate in more detail the proximate and ultimate mechanisms underlying the evolution of alternative reproductive tactics and strategies in social insects.

## Methods

### Ant collection and cultivation

According to detailed field studies by Felke & Buschinger [27], *L. acervorum *in central Spain is restricted to pine forests at elevations above 1500 m. We therefore focused our study on these previously described collecting sites in the Sierra de Albarracin (September 2008 and May 2009) [27] and also identified similar sites at Sierra de Gúdar (May 2009), both province of Teruel, Spain (Table 1). Colonies were housed in the laboratory in three - chambered plastic boxes (10 cm × 10 cm × 3 cm) with plaster floor using standard methods [40]. We kept the ants in incubators in near-natural conditions with ten to twelve weeks hibernation (at 12 h/12 h 5°C/0°C), and thereafter at spring conditions (12 h/12 h 20°C/10°C) for the duration of the behavioral studies [37]. Meteorological data [41] and observations suggest that the ants hibernate for an even longer period in the field. Ants were fed with honey, cockroaches and water twice weekly.

### Behavioral observations and ovary dissections

For the behavioral observations, we chose colonies with four to seven queens (Table 1). All queens were individually marked with 30 to 88 μm thin metal wires (red enameled, black, green, violet and copper) tied between alitrunk and petiole, petiole and postpetiole, and/or postpetiole and gaster.

Observations were started three days after marking and carried out under spring conditions (20°C/10°C). Colonies were directly observed in 20 to 60-min sessions each under a binocular microscope by scan sampling every 5 minutes and in addition by opportunistic sampling [42]. We chose to observe interactions directly as one of the most frequent aggressive interactions in ants, rapid bouts of antennal boxing, is not easily detected on video recordings. We noted the occurrence of all interactions involving queens (antennal boxing, mandible opening, biting, pulling, stinging/smearing, grooming, and trophallaxis, i.e., exchange of liquid food). Overall, we observed the eight colonies for 580 to 1645 min. Rates of behavior were calculated as the frequency of behavior divided by the total length of time a focal colony was observed (hours, Table 1) and the number of queens per colony. They are indicated as behavioral events per hours and individual.

After the observation period, we killed the queens by freezing them at -20°C and dissected their ovaries under a binocular microscope to check for ovarian status. Dissections were carried out as described in [43]. We noted the presence of maturing oocytes, corpora lutea, and sperm in the spermatheca. Ovarian status was classified following [16].

### Statistical analyses

The antagonistic behavior among queens was used to calculate the average dominance index (ADI, [30]) for each queen per colony. For the analysis of relationships between specific behavioral interactions and the future reproductive state of queens we conducted a logistic regression (reproductive status was binary coded: 'reproductive' = 1, 'non - reproductive' = 2). We used the Scheirer - Ray - Hare test [44] as an independent method to compare the predictive power between behavioral interactions and the future reproductive state of queens. All unmated queens and queens with undetermined reproductive state were omitted from analysis. The Mann - Whitney U - test was used to test for differences between queen - queen aggressive behavior and worker - queen aggression. All statistical analyses were carried out in SPSS version 17 and JMP 8.01 (SAS, 2009). Scheirer - Ray - Hare test was performed with Excel version 2007.

### Genetic analysis

In addition to the behavioral studies we extracted genomic DNA from 119 workers out of ten colonies from one sample site in the Sierra de Albarracin (SA 64, SA 65, SA 66, SA 68, SA 70, SA 74, SA 76, SA 78 & SA 88, 12 workers and SA 61, 11 workers) using a CTAB (Cetyltrimethyl ammonium bromide) protocol (modified after [45]). Eleven to twelve workers per colony were genotyped at five polymorphic microsatellite loci that have previously been shown to be informative in this genus: LXAGA1, LXAGA2, LXAGT1 [46], L18 [47], and Myrt3 [48].

PCR conditions were mainly as previously described [45-47] with following annealing temperatures: LXAGA1 at 45°C, LXAGA2 at 50°C, LXAGT1, L18 and Myrt3 at 54°C. Primers were labeled with FAM, HEX, TET fluorescence dyes (Eurofins MWG) and amplification products were analyzed with a capillary sequencer (ABI PRISM 310 Genetic Analyser, Applied Biosystems). We determined allele length using the software GeneScan 3.1 (PE Biosystems). Worker genotypes were used to estimate nestmate relatedness (r ± SE by jackknife over colonies; [49]) with RELATEDNESS 4.2. In addition, Fisher's method implemented in Genepop 4.0 [50] was used to test for linkage disequilibrium between the five microsatellite loci.

## Authors' contributions

AB and JH devised the study and took part in field work; JT did most of the collection and, together with MH, the genetic and behavioral studies and analyzed the data. JT and JH wrote the paper. All authors read and approved the final manuscript.

## Supplementary Material

Additional file 1 **Fighting queens of *L. acervorum *from central Spain**. The movie shows two queens involved in aggressive interactions. They bite, pull and dry to sting each other. At the same time, one queen is attacked by a worker. Click here for file
